# The Networked Interaction between Probiotics and Intestine in Health and Disease: A Promising Success Story

**DOI:** 10.3390/microorganisms12010194

**Published:** 2024-01-18

**Authors:** Maria Skoufou, Christina Tsigalou, Stergios Vradelis, Eugenia Bezirtzoglou

**Affiliations:** 1 Master Program in “Food, Nutrition and Microbiome”, Department of Medicine, Democritus University of Thrace, 68100 Alexandroupolis, Greece; ctsigalo@med.duth.gr (C.T.); svradeli@med.duth.gr (S.V.); 2 Proctology Department, Paris Saint Joseph Hospital Paris, 75014 Paris, France; 3 Laboratory of Hygiene and Environmental Protection, Faculty of Medicine, Democritus University of Thrace, 68100 Alexandroupolis, Greece; 4 Department of Gastrenterology, Faculty of Medicine, Democritus University of Thrace, 68100 Alexandroupolis, Greece

**Keywords:** probiotics, prebiotics, synbiotics, IBD, SCFAs

## Abstract

Probiotics are known to promote human health either precautionary in healthy individuals or therapeutically in patients suffering from certain ailments. Although this knowledge was empirical in past tomes, modern science has already verified it and expanded it to new limits. These microorganisms can be found in nature in various foods such as dairy products or in supplements formulated for clinical or preventive use. The current review examines the different mechanisms of action of the probiotic strains and how they interact with the organism of the host. Emphasis is put on the clinical therapeutic use of these beneficial microorganisms in various clinical conditions of the human gastrointestinal tract. Diseases of the gastrointestinal tract and particularly any malfunction and inflammation of the intestines seriously compromise the health of the whole organism. The interaction between the probiotic strains and the host’s microbiota can alleviate the clinical signs and symptoms while in some cases, in due course, it can intervene in the underlying pathology. Various safety issues of the use of probiotics are also discussed.

## 1. Introduction: Probiotics as Novel Solutions to Old Problems

In the human body, the main site of interactions between microorganisms and the host immune system is the gastrointestinal tract (GIT) which is the largest digestive organ in the human body [1,2]. The human intestine harbors a huge number of microorganisms which belong to large number of species in relation to other parts of the body which are colonized by microorganisms, such as the skin and the upper respiratory systems [3,4,5]. It comprises about 1500 species which progressively colonize the digestive tract starting within minutes of birth by instituting a symbiotic or mutualistic relationship with epithelial and lymphoid tissues [3,5,6,7,8,9]. The interaction between microorganisms and microbiota contributes to the physiological functions of the host while the host provides nutrition and habitat [10]. The role of gut microbiota is essential not only for the degradation and the fermentation of feed but also for the defense against several pathogens either by competing for nutrients and adhesion sites or by secreting antimicrobial peptides [11,12].

This diverse community includes fungi, bacteria, viruses, and bacteriophages, all of which play essential roles in maintaining intestinal homeostasis. The bacteria which are predominately found in the intestinal microbiota belong to the phyla Firmicutes, Bacteroidetes, Actinobacteria and Proteobacteria, mostly colonizing the colon [13]. Intestinal microorganisms significantly affect the state of human health by producing several metabolites from the anaerobic fermentation of exogenous dietary components or from endogenous compounds produced by the host. These microbially-derived metabolites, such as short chain fatty acids (SCFAs), interact with the host cells and influence immune responses [14,15]. This dynamic and complex ecosystem helps the proliferation, growth and differentiation of epithelial cells, thus enhancing the host’s ability to defend against infections and stimulate the immune system [16,17,18]. Numerous experiments conducted on germ-free animals (GF) have elucidated that early colonization of the microbiota is essential for the proper development of immunity. Human-based genetic studies on microbiota demonstrated that an imbalance in the gut microbiota is associated with various inflammatory diseases, liver condition diseases, colorectal cancer, and metabolic disorders [19,20,21,22].

The term “intestinal health” describes the condition and effective operation of the intestines, a component of the digestive system [23]. In the absence of gut microbiota, the intestinal mucosal immune system remains underdeveloped, resulting in reduced numbers of functional regulatory CD4+ CD25+ T cells, and, consequently, a diminished ability to combat pathogenic bacteria [12,24]. Furthermore, the balance between pro-inflammatory interleukin (IL)-17-producing effector T helper (Th17) cells and regulatory Forkhead box P3 (Foxp3+) T cells (Tregs) in the gut requires signals from gut bacteria, and these signals depend on the composition of the intestinal microbiota [25]. For example, GF animals that were colonized with *Bacteroides fragilis* showed a restoration of the balance between Th1 and Th2 cells, which was attributed to the production of polysaccharide A [26]. The general well-being of the intestines, including the small and large intestines, depends on maintaining a healthy intestinal tract [4,27].

Elie Metchnikoff (1845–1916) is accredited with initiating the concept that live microorganisms are beneficial for health. He also proposed the idea of implanting lactic acid bacteria (LAB) such as Lactobacilli from a Bulgarian yogurt culture [28,29]. Probiotics are defined as live microorganisms present in foodstuffs that, when consumed at certain levels as part of nutrition, stabilize the GIT and thereby confer health benefits on the consumer [29,30,31]. Some researchers reported that populations of 10^6^–10^7^ colony-forming units (CFU/g) or CFU/mL in the final product are established as therapeutic quantities of probiotic cultures in processed foods [28,30]. The Food and Agriculture Organization of the United Nations (FAO) and the World Health Organization WHO classify probiotics as live microbes that, when applied in adequate amounts, provide health benefits to host conditions [32,33,34]. Probiotics are usually consumed as a dietary supplement if not as part of the microbiota of foods such as cheese or kefir. According to Liu et al., probiotics are essential for maintaining gut health as they provide vital nutrients and help reduce inflammation [35,36,37]. A healthy and balanced diet plays a crucial role in promoting immunological function, which is essential for defending the body against illnesses and infections. However, immune health can be influenced by a combination of genetic and immunological factors, as well as lifestyle choices, infections, and hormone imbalances, making the relationship between nutrition and immune function complex [34,38] Nowadays, probiotics have attracted researchers across the globe for their widespread beneficial health-promoting properties, especially in irritable bowel syndrome and antibiotic-associated diarrhea [39]. Some studies in inflammatory bowel diseases have suggested that probiotics may have a positive impact on specific aspects of Inflammatory Bowel Disease (IBD) [39,40,41]. However, more research is still needed to fully understand their potential benefits [42]. Several studies have highlighted the significance of probiotics that influence intestinal homeostasis such as intestinal barrier function through Tight Junction (TJ) complex regulation [43].

As the interest in the utility of probiotics grows, researchers are exploring the potential for mixed approaches in treating gut inflammation. In this review article, new trends in probiotics and novel methods of treatment are addressed, and recommendations for the future are proposed. Mixed approaches, which combine probiotics and prebiotics, are called synbiotics (Table 1) and have shown promising results in reducing gut inflammation [44,45]. The idea behind this approach is that by combining different types of beneficial bacteria and food ingredients, an environment is created in the gut that supports the growth of beneficial microorganisms, reduces inflammation, and helps maintain a healthy microbiome [44,45,46].

This review discusses, among other topics, how fermented foods, dairy probiotics, and non-dairy-based probiotics improve intestinal immunity by ameliorating the risk of IBD. The promising biotherapeutic effects of various probiotics on intestinal health are also discussed in this article, and their modulation seems to have a significant effect on inflammatory bowel illnesses. When consumed, probiotics travel through the digestive system and interact with gut microbiota. They colonize the intestine and create a more balanced ecosystem, reducing the prevalence of harmful bacteria or interacting with inflammation sites [47].

**Figure 1:**
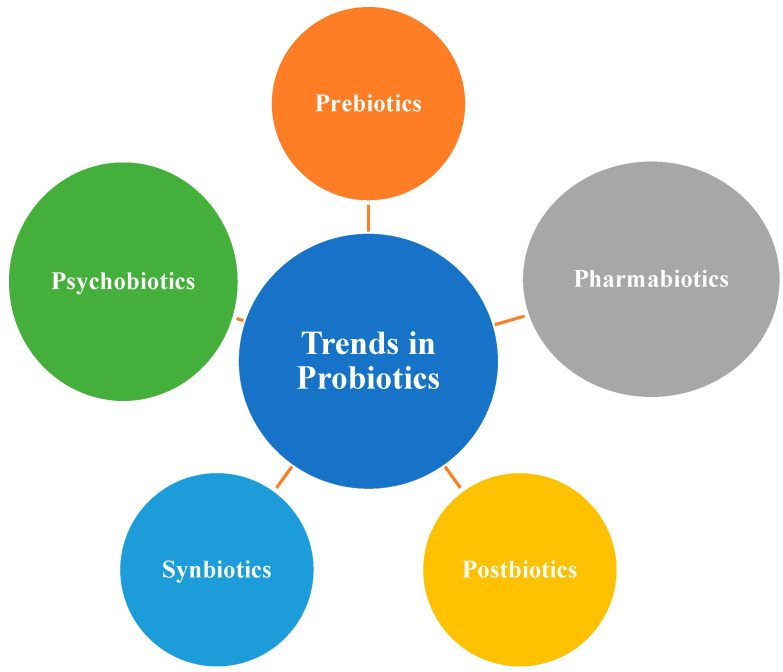
New trends in Probiotics.

New probiotic supplements offer targeted solutions for various gut issues, which can help alleviate inflammation. By taking a mixed approach, one can help to improve the health of the gut, which can have a positive impact on overall health. Further research is needed to fully understand the effects and optimal dosages of these beneficial microorganisms. Precision probiotics, by targeting specific microbial imbalances, can help restore a healthier gut microbiota composition, potentially alleviating inflammation and symptoms associated with inflammatory gut diseases. This personalized approach may enhance the effectiveness of probiotic interventions for inflammatory gut diseases. In the present era of bioengineering, a combination of different probiotic strains, which can be engineered to work together (as engineered microbial consortia), offer enhanced therapeutic effects compared to a single strain (Figure 1). Pharmabiotics are probiotics that can be enhanced with bioactive molecules or drugs to augment their therapeutic efficacy, such as incorporating anti-inflammatory drugs or gut-healing molecules [48].

## 2. Bacterial Probiotic Strains

**Figure 2:**
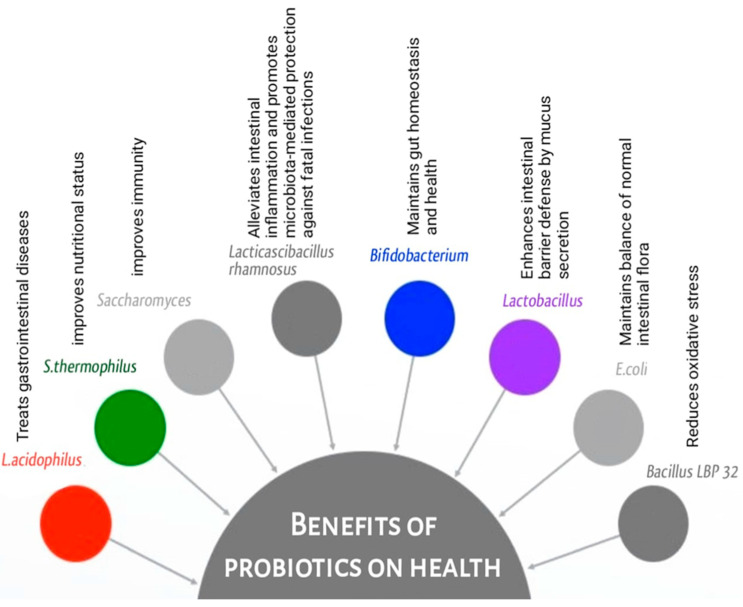
Benefits of probiotics on health.

More than 90% of the gut microbiota include Actinobacteria, Bacteroidetes, Firmicutes, Fusobacteria, and Proteobacteria. Microbial content is highest in the colon [1,22,49]. The composition and diversity of the gut microbiota are closely intertwined with various facets of gastrointestinal health and immune function (Figure 2). Bacterial genera *Bifidobacterium* and *Lactobacillus* are frequently included in probiotic supplements and have been found to have beneficial effects on consumer health. Improvements in gut barrier function, immunological regulation, and metabolite production have all been linked to *Bifidobacterium* and *Lactobacillus* species (Table 2). They may aid in re-establishing a normal microbial population in the digestive tract and have anti-inflammatory effects [50]. *Lactobacillus acidophilus* is a bacterial species naturally found in the intestine and has the ability to produce lactic acid. It is commonly used in probiotic formulations and has been studied for its potential benefits in various gastrointestinal conditions, such as irritable bowel syndrome (IBS) [51]. *Lacticaseibacillus rhamnosus* (previously named *Lactobacillus rhamnosus*) GG (LGG) [52,53] is a widely studied probiotic strain known for its potential benefits in GIT disorders [54]. *Bifidobacterium* species are dominant in healthy individuals and particularly—among other species—*Bifidobacterium breve*, *Bifidobacterium longum* and *Bifidobacterium bifidum* [55]. The latter is one of the most studied strains within this group and has been shown to have potential benefits in improving gut health, supporting immune function, and promoting bowel regularity [56].

The restoration of the imbalanced gut microbiota in cases of IBD is nowadays the focus of scientific and therapeutic research. Numerous strategies attempt to manage the microbiota to improve IBD symptoms and disease outcomes, even though it may be challenging to fully restore the microbiota to a healthy state [47,69,70]. Some beneficial bacteria, like *Lactobacillus* and *Bifidobacterium* species, are commonly known for their positive effects on the digestive system, but emerging research suggests they may also have other health benefits (Figure 2). The most common probiotics include members of the family Lactobacillaceae, *Streptococcus* spp., *Enterococcus* spp., and *S. boulardii* [71,72]. *S. boulardii* is a probiotic yeast that has shown effectiveness in the treatment of certain GIT disorders, including inflammatory gut diseases and recurrent *Clostridioides difficile* colitis [73,74]. It is often used in conjunction with bacterial probiotics. *Streptococcus thermophilus* is commonly used in combination with other probiotics or as a starter culture in dairy fermentation. It has been studied for its potential health benefits [58,62,75]. *E. coli* Nissle 1917 is a specific strain of *Escherichia coli* which has demonstrated probiotic properties and has been studied for its efficacy in treating various gut disorders [63,64] (Table 3). Individuals with IBD have been found to have lower than normal levels of *Faecalibacterium prausnitzii*, suggesting the possible protective role in intestinal health [76]. *Akkermansia muciniphila* is a bacterium that lives in the mucus layer of the gut lining and degrades mucin. Evidence suggests that having *A. muciniphila* in the gut can lead to a more favorable microbiome, better barrier function, and a lower risk of metabolic diseases [77,78] and hence its therapeutic applications in the fields of obesity, endocrine disorders, and inflammatory bowel disease have all been the focus of research [77,78,79].

**Figure 3:**
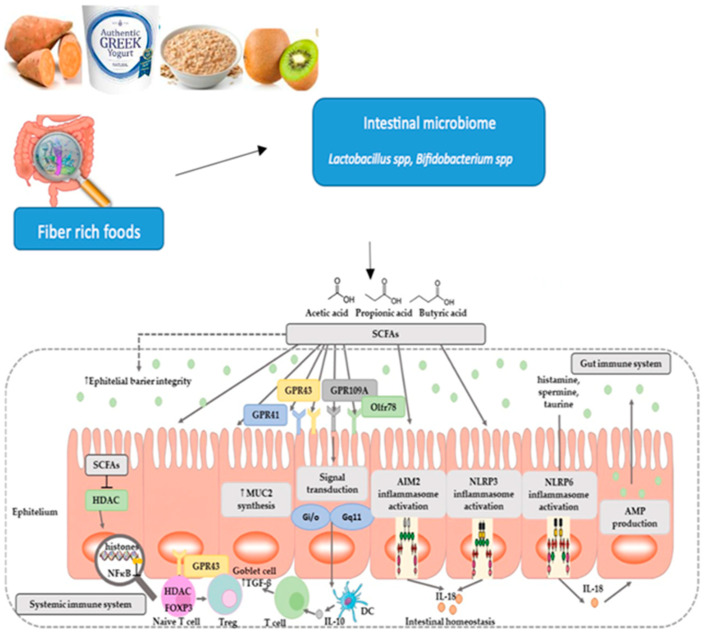
Fiber-rich foods and the role of SCFAs.

What all the above-mentioned intestinal microbiota members have in common are at least three major functions: (i) they inhibit the growth of pathogens by competing with them for food and shelter, or even kill them by secretion of bacteriocins, (ii) they digest host diet and produce essential metabolites like vitamins, amino acids, SCFAs and many more which are necessary for the healthy as well as for the diseased individual and (iii) they contribute to the maturation of epithelioid cells both structurally and functionally, thus strengthening the overall intestinal immune system (Figure 3) by reinforcing the intestinal barrier, which is often disrupted in patients with IBD [80,81].

Antibiotic treatment, widely used in IBD patients, can also alter the population of normal microbiota, and thus alter immunity. The microgram population affects the digestive gut of higher animals by producing its colonies in numbers which eventually damage the functional structure of the gut [80]. This leads to a complex interplay of factors influencing microbiota-associated chronic inflammation in health [22,82]. The gut microbiota can utilize several metabolic pathways which other bacteria cannot, such as bile metabolites [83,84]. Also, lactic acid produced by various *Lactobacillaceae* species inhibits pathogen growth in the gut [85]. The effect of such processes may influence the outcome of an infection or of a bacterial imbalance. Gut microbiota has been implicated in the pathogenesis of inflammatory diseases [80,81,82,83,84,85,86].

## 3. Probiotics and Their Metabolites

**Figure 4:**
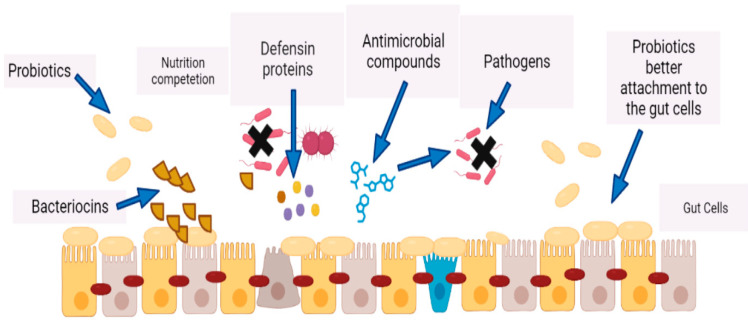
Improved gut immunity by probiotics via production of bacteriocins, defensins, and antimicrobial compounds. There is a nutrition competition between probiotics and the pathogens; probiotics provide better attachment to the gut cells and create a barrier against pathogens.

Various metabolites produced by probiotics including secreted proteins (extracellular proteins), indole, extracellular vesicles, short chain fatty acids (SCFAs) and bacteriocins have the capability to protect the intestinal epithelial barrier by interacting with some receptors [87,88], by killing directly the infectious bacteria [87,88], by directly promoting mucus secretion by goblet cells, increasing thus the secretion of antimicrobial peptides, or by enhancing the expression of tight junctions [87,88,89] (Figure 4). Probiotics have the capability of inhibiting inflammation via several mechanisms which affect the intestinal microbiota [57]. A study performed by Yan et al. demonstrated that *Lcb. rhamnosus* (LGG)-derived soluble protein p40 can prevent and treat experimental colitis by relying on epidermal growth factor receptors [90]. Moreover, probiotics are also capable of producing cytokines by affecting the epithelial cells and have anti-inflammatory effects. Zhang et al. confirmed that LGG reduced TNF-a, induced IL-8 production by affecting the NF-kB pathway in Caco-2 cells [91]. Similarly, Madsen et al. reported that culturing epithelial cell monolayers with probiotics can avert changes in epithelial permeability caused by pro-inflammatory cytokines TNF-a and IFN-g [92]. Probiotics stimulate the production of defensin from intestinal crypts, thereby regulating the proliferation of normal micromicrobiota in the crypts and persuading the morbidity of IBD in these areas [93,94]. Bacteriocins can be used as antibacterial peptides competent to promote to a dominant position the producing bacteria in their niche and so securing a competitive advantage for probiotics [95]. Bassaganya Riera et al. reported that the conjugated linoleic acid (CLA) produced by probiotics can retain intestinal homeostasis by inducing and activating peroxisome proliferator-activated receptor gamma and delta (PPAR-γ or PPARg and PPAR-δ or PPARd), thus preventing the progression of and improving the lesions of inflammatory enteritis [96]. Lactococcin (a protease secreted by *Lacticaseibacillus paracasei* subsp. *paracasei*, previously named *Lactobacillus paracasei*) can degrade some pro-inflammatory chemokines including CXC chemokine ligand 10 to inhibit the staffing of inflammatory cells to mucosal tissues and prevent colitis in mice [97]. CLA refers to positional and geometric isomers of Linolic Acid (LA), and some research studies also suggest that CLA is a type of healthy fat that has been shown to have cancer-fighting properties [98,99]. It is commonly found in dairy products like milk and yogurt. Organic acids are produced by probiotics during the fermentation process [100]. They promote the growth of other beneficial bacteria in the gut and help maintain a healthy pH balance. SCFAs are produced by gut bacteria during the digestion of fiber. They can improve intestinal barrier function, reduce inflammation, and promote healthy immune function [31,101]. Bile acids, which are involved in the metabolism of fats, are produced by the liver and can be modified by gut bacteria [102].

Another study by Segawa et al. confirmed that polyphosphate derived from Lactobacillus brevis had a protective effect on epithelial cells by activating mitogen protein kinase p38 [103]. The human gut microbiota is also capable of producing essential vitamins that can neutralize the effects of some toxic compounds such as pyrolysates [104]. There is a direct relation of host metabolism with microbial metabolites which bind to specific host membranes/receptors [105]. Among these metabolites, the most important are described in Table 4. Many of the essential functions of the host’s body as well as its maintenance are associated with gut microbiota; for example, nervous system, intestinal development and appetite regulation [22,83,106]. When consumed, probiotics travel through the digestive system and interact with gut microbiota. They colonize the intestine and create a more balanced ecosystem, reducing the prevalence of harmful bacteria (Figure 4). Similarly, Levit et al. in their study compared a riboflavin-producing LAB strain with commercially available vitamin supplements in a TNBS-induced mouse colitis model, and confirmed that soyabean milk fermented by *Lpb. plantarum* CRL2130 can produce riboflavin which has an anti-inflammatory effect [107]. Therefore, microorganism-produced riboflavin and other vitamins may be used as a new tool for probiotics to treat IBD. Some research studies have found that other unidentified compounds secreted by *E. faecalis* and *Lcb. paracasei* can inhibit the activation BF-kb and protect the tissues of patients with IBD from experimental colitis or ongoing inflammation [108,109]. A recent study by Pujo et al. showed that long-chain fatty acids produced by *E. coli* Nissle 1917 (EcN) can bind to and activate PPARg to exert anti-inflammatory effects, thereby inhibiting DSS-induced colitis in mice [110]. Butyrate is perhaps the most studied SCFA with significant reported evidence showing its anti-inflammatory and anti-carcinogenic effects [111]. It also has a role in overall gut health because it is the primary energy source for colonic mucosa [112,113,114].

## 4. Probiotics and Food Products

**Figure 5:**
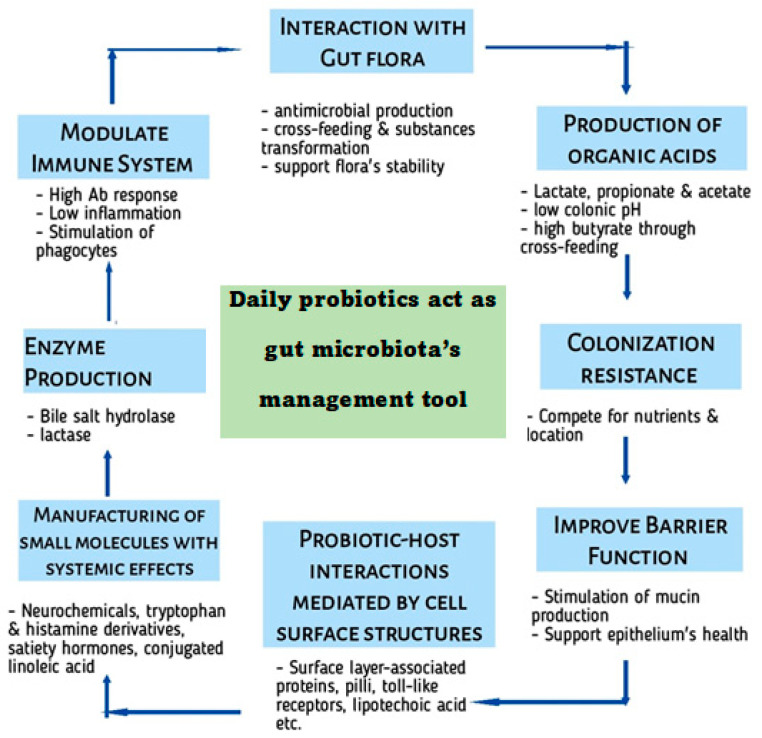
Role of dairy food probiotics in the management of gut microbiota.

Probiotic strains commonly found in dairy products such as yogurt, kefir, and cheese are *Lb. acidophilus*, *Lcb. casei*, *Lpb. plantarum*, *Lcb. rhamnosus*, *S. thermophilus*, and *Bifidobacterium lactis*. *Lactobacilli* shows antirotaviral and antibacterial activity by promoting metabolites like bacteriocins, non-bacteriocins and lactic acid [131,132,133,134,135]. Thus, they promote a healthy balance of gut bacteria, improve lactose digestion and support digestive health (Figure 5). *Lcb. rhamnosus* has been studied for its potential benefits in supporting the immune system and promoting gastrointestinal health [136].

Non-dairy probiotics can be an excellent option for individuals who are lactose intolerant, followed by those who adhere to a vegan or dairy-free diet, or prefer non-dairy sources. Non-dairy probiotic strains include more lactobacillus strains compared to dairy products. Some of them are *Lb. acidophilus*, *Lcb. casei*, *B. lactis*, *Lpb. plantarum*, *B. thermophilus*, *B. faecium*, *E. faecium*, *S. boulardii*, and *Lcb. rhamnosus*. Non-diary probiotic supplements containing *L. acidophilus* produce lactase enzyme, having two domains, and exhibit catalytic activity toward beta-glucopyranosides and beta-galactopyranosides, with a preference for hydrophilic aglycones present in lactose and cellobiose in one domain and hydrophobic aglycones in phlorizin and glycosylceramides in the other domain [137,138]. The enzymatic hydrolysis of lactose by this enzyme results in the production of D-glucose and D-galactose, which are essential for the proper absorption and utilization of these sugars by the body. Foods like sauerkraut, kimchi, and pickles are examples of fermented vegetables that contain live cultures of beneficial bacteria [139]. Similarly, fermented soy products, kefir, and probiotic supplements in the form of capsules, tablets, and powders are examples of non-dairy probiotic sources. Fermented soy products like tempeh and miso are rich in probiotics [42]. Non-dairy kefir provides probiotics and can be a suitable option for individuals avoiding dairy products. IBD is a risk factor for lactose intolerance and other functional intestinal conditions [140]. The inflammation in the gut can damage the cells that produce lactase, leading to a reduced ability to digest lactose and causing GIT symptoms. In such conditions, non-dairy probiotics should be thought of as an alternative in order not to aggravate intestinal symptoms [141]. There is a misconception that Lactobacilli is present only in dairy products, but studies show the presence of *Lactobacillus* strains in dairy as well as in nondairy probiotics [142,143].

## 5. Exploring the Role of Probiotics in Managing Intestinal Diseases

Inflammatory bowel diseases are associated with an imbalance in the gut microbiota [144]. This dysbiosis affects the intestinal barrier permeability which promotes the exposition to luminal content and triggers an immunological response that leads to intestinal inflammation. The mucosal innate immune system must differentiate between commensal bacteria and harmful germs to preserve intestinal homeostasis without triggering local inflammation [22,42,112]. These diseases significantly impact patient lifestyle quality and require long-term management and enhancement. Below is a detailed explanation of how gut diseases affect the microbial population.

### 5.1. Crohn’s Disease

Crohn’s disease can impact various segments of the digestive tract, often in a scattered pattern, and it is distinguished by inflammation that extends through the entire wall of the affected area. This condition can give rise to complications like fibrotic strictures, fistulas, and abscesses. In this dysimmune-based disease, the defense mechanism mistakenly attacks the intestinal lining, leading to inflammation. This chronic inflammation can disrupt the balance that is decrease in the variation of microorganisms and increase in specific bacterial taxa [145]. There is a decrease in beneficial bacteria such as Firmicutes and Bacteroidetes and an increase in potentially harmful bacteria like Enterobacteriaceae [146,147]. This disease exhibits varied involvement within the gastrointestinal tract, with the highest prevalence seen in the ileocolonic region (38%), followed by the ileum (47%) and colic (15%) regions. In terms of disease behavior, Crohn’s disease is predominantly characterized by inflammatory behavior (63%), while stenosing (22%) and fistulizing (15%) behaviors are also common. The dysbiosis observed in Crohn’s disease can further contribute to inflammation and disease progression. Dysbiotic microbiota may impair the gut’s barrier function, increase intestinal permeability, and promote the production of pro-inflammatory molecules. This, in turn, perpetuates the inflammatory response and leads to the exacerbation of symptoms [147]. Some studies testing probiotics in Crohn’s disease have produced negative results so far [147,148].

### 5.2. Ulcerative Colitis

Ulcerative colitis (UC) exclusively affects the colon and involves superficial inflammation of the mucosal lining that extends continuously from the distal to the proximal regions. This condition can result in ulcerations, severe bleeding, toxic megacolon, and fulminant colitis. Similar to Crohn’s disease, the immune system in ulcerative colitis mistakenly targets the gut lining, resulting in chronic inflammation. This inflammation can also disturb gut microbiota composition. Ulcerative colitis, on the other hand, primarily affects the colon. The distribution of ulcerative colitis includes proctitis (12%), left colitis (32%), and pancolitis (56%), reflecting the extent of colon involvement [149]. In ulcerative colitis, dysbiosis is typically marked by reduced microbial diversity and alterations in specific bacterial groups [150]. There is a decrease in beneficial bacteria such as Firmicutes, Bacteroidetes, and *F. prausnitzii*, which is a butyrate-producing bacterium with anti-inflammatory properties [76]. Concurrently, there is an increase in Proteobacteria, a phylum that includes potentially pathogenic bacteria. The dysbiosis observed in ulcerative colitis may contribute to the perpetuation of inflammation. Imbalances in the microbial population can disrupt the formation of essential metabolites like SCFAs, impair the gut barrier function, and modulate immune responses, leading to sustained inflammation in the colon [12,151]. Restoring the gut microbiota in IBDs involves various approaches, such as dietary modifications, probiotics, prebiotics, antibiotics, and fecal microbiota transplantation (FMT) [152,153]. Unlike Crohn disease, probiotics were effective in UC. Randomized controlled trials (RCTs) testing probiotics have demonstrated the effectiveness of product VSL#3 in preventing pouchitis, as well as *E. coli* Nissle 1917 in preventing the relapse of ulcerative colitis [154]. A high dose of probiotic therapy (VSL#3) is administered once daily for maintaining remission in recurrent or refractory pouchitis. VSL#3 is a combination of eight strains, including *B. breve*, *B. longum*, *B. infantis*, *Lpb. plantarum*, *Lb. acidophilus*, *Lcb. casei*, *Lb. delbrueckii* subsp. *bulgaricus*, and *S. thermophiles*. Additionally, long-term sustainability and maintenance of the restored microbiota are areas of ongoing investigation [112,155].

### 5.3. Infectious Colitis

Infectious colitis (IC) refers to inflammation of the colon caused by infection, often resulting from bacteria, viruses, or parasites. During an episode of infectious colitis, there can be a transient disruption in the gut microbial population. The specific impact on the gut microbiota during infectious colitis depends on various factors, including the infecting agent and the individual’s immune response [156]. However, in many cases, there is a temporary alteration in the relative abundance of different microbial species. Once the infection is treated and inflammation subsides, the gut microbiota tends to recover and return to its pre-infection state. However, in some cases, the infection and associated inflammation can cause lasting changes to gut microbiota composition, provoking symptoms that are categorized as post-infectious irritable bowel syndrome and may include abdominal discomfort, bloating, and alternation between diarrhea and constipation [157]. It is important to note that the exact mechanisms underlying the relationship between gut inflammation and gut microbiota are still being studied. The bidirectional interactions between the gut microbiota and the host immune system are complex and multifaceted. Further research is needed to understand the specific cause and effect relationships and identify potential therapeutic interventions to modulate gut microbiota in inflammatory gut diseases. Overall, inflammation diseases of the gut can disrupt the balance and composition of gut microbiota, leading to dysbiosis. This dysbiosis, in turn, may contribute to perpetuation of inflammation [158].

As far as *C. difficile* colitis is concerned, a colitis caused by antibiotic consummation, although *S. boulardii* has been effective in treating recurrent infection, the evidence is still insufficient to recommend probiotics, according to the latest European guidelines of 2021 [159].

In the case of recurrent *C. difficile* colitis, fecal microbiota transplantation (FMT) from a healthy individual to a patient has demonstrated effectiveness [160]. It is now considered a recommended second-line treatment for this rare and serious condition, with an efficacy of over 80% [161]. Fecal microbiota transplantation (FMT) involves administering a preparation of fecal matter from a healthy individual to a patient affected by a condition related to an alteration of the intestinal microbiota, exerting therapeutic effects. Given the involvement of the microbiota, researchers are investigating FMT in the context of inflammatory bowel diseases (IBD), and patients hope that this approach could serve as an alternative to immunomodulatory drugs and/or a curative treatment for their condition. Open trials in subjects with IBD or a combination of IBD and *C. difficile* infection have suggested effectiveness in IBD cases [162,163], yet more research should be conducted to obtain safe and conclusive results.

### 5.4. Celiac Disease

Celiac disease is an autoimmune disorder triggered by the ingestion of gluten, a protein found in wheat, barley, and rye. In individuals with celiac disease, the immune system reacts to gluten, leading to inflammation and damage to the small intestine. This inflammation can impact gut microbiota composition. Studies have shown that individuals with celiac disease often exhibit alterations in gut microbiota compared to non-celiac individuals [164]. Dysbiosis in celiac disease is characterized by reduced microbial diversity, decreased levels of beneficial bacteria such as *Bifidobacterium* and *Lactobacilli*, and increased levels of potentially pathogenic bacteria. The dysbiosis observed in celiac disease may contribute to inflammation and intestinal damage. The altered gut microbial composition can affect the solidarity of the intestinal barrier, influence immune responses, and potentially modulate the presentation of gluten antigens, thereby influencing disease progression and symptoms [165]. Adherence to a strict gluten-free diet is the primary treatment for celiac disease. This dietary intervention has been shown to lead to improvements in gut microbial composition. Additionally, specific probiotic strains and dietary fiber may help restore dysbiosis in individuals with celiac disease, although further research is needed to establish their efficacy [166,167,168]. Researchers have conducted analysis to explore the potential role of a specific probiotic preparation, VSL#3 (see above, in ulcerative colitis). The study aimed to investigate how VSL#3 can reduce the toxic properties of wheat flour during prolonged fermentation. The findings revealed that VSL#3 exhibited a high level of effectiveness in hydrolyzing gliadin polypeptides when compared to other commercial probiotic products [169,170]. Despite ongoing research, a consensus on shifts in bacterial composition, particularly at the species level, remains elusive [167,168]. Consequently, forthcoming studies should prioritize in-depth microbiota characterization to explore its potential benefits for gut health.

### 5.5. Irritable Bowel Syndrome (IBS)

Irritable bowel syndrome, a complex and multifactorial GIT disorder, is characterized by symptoms related to the way the gut functions rather than by structural or biochemical abnormalities. The main key point is abdominal pain and changes in bowel habits partially due to dysbiosis. Research suggests that IBS patients often face dysbiosis that is directly related to reduced microbial diversity, increase in specific bacterial taxa, and alterations in the production of probiotic metabolites [171,172]. The dysbiosis in IBS can contribute to the symptoms experienced by individuals. Altered gut microbiota composition and function can impact gut motility, visceral sensitivity, immune responses, and the integrity of the gut barrier, all of which are involved in the pathophysiology of IBS [173,174]. *Lactobacilli* produce lactic acid and help create an acidic environment leading to the inhibition of growth of harmful bacteria. Apart from that, they are involved in the removal of pathogens by competing for the sites of adhesion and nutrient intake. These bacteria promote mucin production and regulate the production of TJ proteins, preventing the entry of toxins and pathogens [172]. Other bacteria including strains of *Streptococcus* and *Enterococcus* have the ability to ferment dietary fibers, producing SCFAs and thus helping in maintaining the health of gut epithelium. These can also interact with immune cells by influencing the production of cytokines which regulate immune response. Some *Enterococcus* spp. possess bile salt hydrolase activity, allowing them to metabolize and modify bile salts. This can have implications for bile acid homeostasis and overall gut health [175]. The management of irritable bowel syndrome (IBS) often involves dietary modifications, probiotics, and other interventions targeting gut symptoms. Probiotics, particularly certain strains like *Bifidobacterium* and *Lactobacilli*, have shown promise in restoring dysbiosis and alleviating symptoms in some individuals with IBS. However, response to interventions can vary, and personalized approaches may be necessary [38].

### 5.6. Colorectal Cancer (CRC)

Studies have shown that individuals with colorectal cancer often exhibit dysbiosis in the gut microbiota compared to healthy individuals [176]. Dysbiosis in CRC is characterized by changes in microbial diversity, alterations in specific bacterial taxa, and an imbalance in beneficial and harmful microbes. The dysbiosis observed in colorectal cancer may contribute to tumor development and progression. Altered gut microbial composition can influence immune responses, the production of metabolites, and the integrity of the gut barrier, all of which can impact the inflammatory environment and potentially promote carcinogenesis [105]. Dysbiosis in colorectal cancer (CRC) is an area of active research, while the restoration of dysbiosis in CRC is not yet well-defined. Some studies have shown that dietary interventions, such as increased intake of dietary fiber and specific polyphenols, may positively influence gut microbiota composition [177,178,179]. To study the impact of these interventions on dysbiosis restoration in CRC, further research is required. It is important to note that the field of microbiota restoration is rapidly evolving, and ongoing research aims to better understand the complex interactions between gut microbiota, diseases, and potential therapeutic interventions. Individualized approaches based on a person’s unique characteristics and disease presentation are likely to be important for optimizing dysbiosis restoration in these diseases. Consulting with healthcare professionals or specialists knowledgeable in the specific disease is crucial to determine appropriate treatment strategies [180].

## 6. Exploring the Axis Administration of Probiotics—Human Gut (In Health and Diseases)

### 6.1. Several Metabolites Production

Dietary factors play a significant role in the prognosis of gastrointestinal diseases by affecting the gut microbial microbiota composition and its function [181]. These conditions have persistent inflammation that might upset the equilibrium of the gut microbiota and bring about dysbiosis. Inflammatory bowel disease is characterized by a reduction in anti-inflammatory microorganisms such as Bifidobacteria and *Lactobacilli*. Simultaneously, dangerous bacteria like some types of *E. coli* and Proteobacteria can flourish. These shifts in the microbial community could have a role in the perpetuation of inflammation and the development of IBD [69,182]. Increased inflammation and susceptibility to infection can result from dysbiosis, which can impair immunity and the intestinal barrier [182]. Metabolites and short-chain fatty acids, which help keep the gut healthy and moderate immunological responses, can be affected as well [183,184].

Fermented foods have been consumed for centuries and are known for their potential benefits to intestinal health. They are rich in beneficial bacteria, enzymes, and other bioactive compounds that can support a healthy gut microbiome. Individuals with digestive issues such as bloating, gas, or lactose intolerance must use fermented foods as the beneficial bacteria in fermented foods aid in the breakdown, bioavailability, and absorption of certain compounds, including vitamins, minerals, and antioxidants [185]. This contributes to overall nutrient status and supports various bodily functions. A healthy gut barrier reduces the risk of inflammation and immune system activation by inhibiting the entry of toxic substances into the bloodstream. Some studies suggest that fermented foods may have antagonistic inflammatory effects. The presence of certain probiotic strains and bioactive compounds in fermented foods can modulate the immune response and help to reduce gut inflammation [186]. To stop Shigella from being harmful in mice models, the effectiveness of a whey pearl millet-barley probiotic beverage has been studied [187]. Reviewing the relationship between probiotics (*S. boulardii*, *Lcb. rhamnosus*, and the trio of *Lb. acidophilus*, *Lcb. casei*, and *Lcb. rhamnosus*) and *C. difficile* infection, increasing evidence points to the safety and utility of probiotics as a primary preventative measure [188]. *S. boulardii* is a non-pathogenic yeast that has been extensively studied for its probiotic properties. It has been shown to help restore the gut microbiota balance, support digestive health, and reduce the risk of antibiotic-associated diarrhea and *C. difficile* infection [189]. A meta-analysis of randomized clinical studies has shown that probiotics impact antioxidant status; oxidative stress is linked to the etiology of numerous diseases [190].

### 6.2. Impact of Diet on Gut Microbiota

Diet is increasingly acknowledged as a key aspect in the management of symptoms and support of intestinal health in inflammatory diseases. The gut microbiota, intestinal inflammation, and general digestive health are all affected by dietary choices [191,192,193]. Foods with anti-inflammatory qualities are prioritized, while those with pro-inflammatory properties are avoided or consumed in moderation, as part of an anti-inflammatory diet. This typically entails eating a lot of anti-inflammatory foods including fruits, vegetables, whole grains, lean proteins (like fish and chicken), healthy fats (like olive oil and avocados), and spices and herbs. Consumption of processed foods, added sugars, trans fats, and possible trigger foods (which might vary from person to person) should be limited or avoided. It has been demonstrated that higher ultra-processed food intake is associated with higher risk of IBD [194,195]. Prebiotics (non-digestible fibers) enhance the multiplication and functions of normal microbiota in the gut, while probiotics, when taken in enough amounts, also provide health benefits [196]. Adequate fiber consumption is essential for preserving digestive tract health. Oats, legumes, and fruits are all good sources of soluble fiber, which aids in digestion and encourages the growth of good bacteria in the gut. Whole grains and vegetables are good examples of insoluble fiber, and they help promote regular bowel motions by increasing stool volume. Fiber and prebiotic interventions have emerged as potential therapeutic strategies for IBD, as they can modulate the gut microbiome and promote the growth of beneficial bacteria [197]. The efficacy of these interventions lie in improving clinical outcomes and reducing disease activity in pediatric patients with IBD [77]. For instance, a randomized controlled trial showed that a high-fiber diet significantly reduced disease activity in pediatric patients with Crohn’s disease [198]. Similarly, a meta-analysis of randomized controlled trials found that prebiotic interventions improved symptoms and reduced inflammation in patients with ulcerative colitis [199]. Other novel therapies, some still under research, include stem cell therapy, gene therapy, and fecal microbiota transplantation [200]. Dysbiosis in various gut inflammatory diseases can be restored by making use of probiotics. Probiotic bacteria produce many antimicrobial substances that play a vital role in preventing pathogen colonization. Several studies on in vitro and in vivo models suggest the anti-inflammatory potential of certain probiotic strains, especially *Lactobacilli* [35]. The immunomodulatory properties of this strain significantly reduce inflammation through immune cells [201].

## 7. Probiotics/Symbiotics/Postbiotics

Probiotics, prebiotics, and symbiotics are all part of the functional food concept promoting intestinal health. Prebiotic substances are unable to be digested and resistant to being broken down by stomach acid and digestive enzymes in the human gastrointestinal system, encouraging the development and activity of good bacteria [202]. Consuming prebiotics may therefore benefit the host’s health. According to this, resistant starch and oligosaccharides, which are carbohydrate molecules originating from plants, are the major sources of prebiotics that have been found. Specific oligosaccharide sources that have been shown to promote the activity and expansion of advantageous bacterial colonies in the gut include fructans and galactans. Galactans are made up of galacto-oligosaccharides, whereas fructans are made up of fructooligosaccharides and inulin. The latter, as well as fructo- and galacto-oligosaccharides (FOS and GOS, respectively), are all examples of prebiotic chemicals. Despite starch being resistant, it selectively stimulates the growth and activity of beneficial bacteria in the colon, such as Bifidobacteria and Lactobacilli [35]. By promoting the growth of these beneficial bacteria, prebiotics contribute to healthy gut microbiota and support overall intestinal health. Prebiotics have the potential to help repair dysbiosis in inflammatory bowel disease by creating an environment favorable to the growth of beneficial bacteria [203,204]. Improvement in symptoms and changes in gut microbiota composition have both been linked to prebiotic supplementation in inflammatory bowel disease (IBD), but more research is needed to establish the best kinds and amounts of prebiotics to use. Antibiotics, probiotics, and, more recently, prebiotics and symbiotics may have considerable impacts when used therapeutically to alter bacterial ecology. Similarly, probiotics and prebiotics modulate the microbiome and improve clinical outcomes in patients with IBS [205].

Synbiotics combine both probiotics and prebiotics. They are products that contain both live beneficial microorganisms and the specific prebiotic fibers that support their growth and activity. Probiotics and prebiotics are combined to create complementary synbiotics, each of which acts independently, and which has been proven to have clinically significant health benefits [29,182,206]. The majority of synbiotics sold commercially and utilized in clinical studies are of complimentary variety. On the contrary, synergistic synbiotics imply that additional microorganisms are specifically activated or that the related substrate boosts their persistence or activity [207]. Even though various novel substances have been proposed in recent years based on this theory, comparatively few of them have been tested for symbiotic synergism. By combining probiotics and prebiotics, synbiotics aim to provide a synergistic effect, enhancing the survival and effectiveness of the probiotic strains. Synbiotic supplements offer a convenient way to combine probiotics and prebiotics to improve gut health.

Postbiotics, on the other hand, offer a unique therapeutic avenue. When probiotic bacteria absorb prebiotics, they generate postbiotics, which are bioactive substances. Research suggests that postbiotics can independently exhibit beneficial effects on the gut immune system and inflammation modulation [208]. Butyrate and SCFAs can stimulate regulatory T cells in intestine and thus boost up immunity. The issue of immunity can also be controlled by utilizing postbiotics derived from the growth or fermentation of probiotic bacteria, like *Lactobacilli*, *Enterococcus*, *Streptococcus* and *Bifidobacterium*, thus making it possible to achieve therapeutic benefits without the need for consumption of live bacteria [209]. Postbiotics come in a variety of forms like fatty acids with a short chain, lipopolysaccharides, exopolysaccharides enzymes, cell wall pieces, bacterial lysates, supernatants devoid of cells, and a combination of substances produced by bacteria and yeast metabolites, including vitamins and amino acids [184,203]. Postbiotics have been linked to several additional and recently discovered health advantages like allergy control and others. According to research on individuals with eczema, the severity of the illness was greatly lessened by taking a postbiotic supplement for 8–12 weeks [208,210]. Furthermore, these probiotics suppress hunger signals and lead to weight loss; they play an important role in reducing the risk of cardiovascular diseases and have anti-tumor activities as they inhibit the growth and spread of the cancerous cells [211]. Probiotics and symbiotic supplementation improve glutathione levels as the body’s antioxidant status biomarkers [212]. *Bifidobacterium* strains (*B. lactis* and *B. Bifidum*) and *Lactobacilli* (*Lb. acidophilus* and *Lcb. casei*) have been proven to regulate microbial brain shafts to improve memory defects, brain neurons, and synaptic damage in older mice [213]. New biotherapeutic ways have been opened by using probiotic strains (*Lb. delbruckei* spp. *bulgaricus* and *S. thermophilus*) in cancer treatments [214].

## 8. Selection Criteria of Probiotics in Food

Strict criteria for the selection of probiotics in food have been established concerning their safety, their technological assimilation in the production process, their performance against adverse conditions and finally their impact on the health of the consumer (Table 5). Needless to stress the fact that for a strain to be characterized as probiotic, it must satisfy all four criteria.

## 9. Probiotics and Immune System

Probiotics play a vital role in maintaining bodily equilibrium and preventing/treating diseases in the host. They accomplish this through multiple mechanisms [218,219,220]. Some of the modes they can impact the local immunity system are the following:

- Restoration of Gut Microbiota Homeostasis: By restoring healthy gut bacteria, probiotics can increase resistance to pathogens and stimulate the immune system.
- Modulation of Intestinal Barrier Function: Probiotics can improve gut barrier function and reduce intestinal permeability.
- Production of Short-Chain Fatty Acids: Short-chain fatty acids produced by probiotics regulate the immune system, have an antimicrobial effect and an anti-inflammatory effect.

While the local immunity system focuses on the gut, the inner immunity system operates throughout the body. In this complex network of cells, tissues, and organs, all parts work together to maintain health and to protect against diseases. Probiotics can help by boosting the immune system, reducing inflammation, and even protecting against infections [220,221]. Probiotics can benefit the inner immunity as follows:

- Boosting Immune Cells: Probiotics stimulate the production of immune cells that help defend the body against harmful pathogens.
- Reducing Inflammation: Research suggests that probiotics can help reduce inflammation throughout the body, which is linked to many chronic diseases.
- Protecting Against Infections: Probiotics have been shown to help reduce the risk of infections and may even be effective in treating certain types of infections.

**Figure 6:**
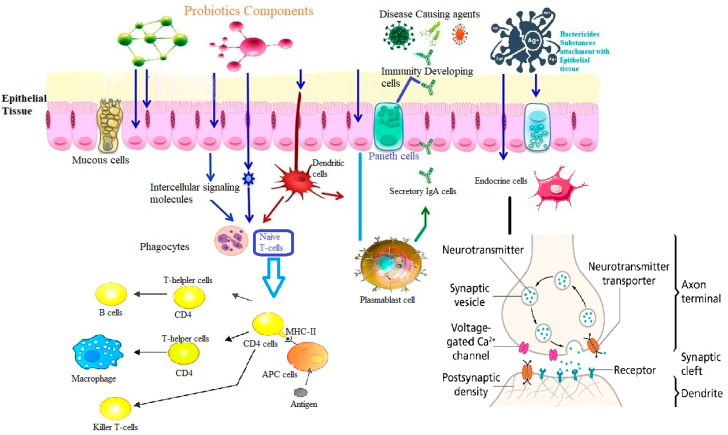
Probiotics and immune system of the gut—Shortline description of the immune—modulatory activity of probiotic: Probiotic components are stimulated through intercellular signaling or through neuro-transmitters the proliferation and chemiotaxis of immune cells series such as dendritic cells (DCs), macrophages, CD4, B and T lymphocyte or generally antigen-presenting cells (APCs). Specialized cells (Microfold-M cells) located in the epithelium overlying Peyer’s patch, receive the antigens and then, with the help of probiotics, they are transferred to the DCs, where the activation process of CD8+/CD4+ naïve T cells and direct helper T cell responses towards Th1, Th2, Th17, or regulatory patterns, is triggered. Once disease-causing agents appear in the GIT, probiotic bacteria are involved in the production of antibodies by plasm-blast cells (or short-lived plasma cells) and activate the Paneth cells in the Lieberkuhn crypts to produce antimicrobial peptides. In addition, probiotic bacteria have the ability to stimulate the production of IgAs in the intestinal lumen, contributing to the enhancement of mucosal and systemic immunity. Finally, stimulation and subsequent signaling of intestinal endocrine cells (EECs) by probiotics can be observed. Enteroendocrine cells produce various hormones which are stored in vesicles within the EECs. The release of these hormones is a regulated process that involves membrane depolarization and calcium influx into the cells.

These interactions involve components such as dendritic cells (DCs), interleukins (ILs), and heat shock proteins (HSPs) [221,222], as depicted in Figure 6.

## 10. Therapeutic Interventions Based on Probiotics

Modifying the gut microbiota may offer an additional strategy for illness prevention and health maintenance. In addition to reducing intestinal inflammation and promoting the production of immunoglobulin A (IgA), probiotic therapy can help stabilize the gut by strengthening the intestine’s immune system [222,223]. Most commercially marketed probiotics make health claims like restoring a healthy bacterial habitat, preventing disease, or improving health somehow, thus lowering the chances of disease onset [224]. *Lactobacilli* as probiotic strains inhibit gastroenteric and foodborne pathogens and food spoilage fungi on probiotics [225]. For example, *Lb. acidophilus* inhibits the growth of common gut pathogen *Helicobacter pylori* [226]. Additionally, the sea buckthorn and apple juice-based probiotic fortifies food substrates to alter the pathogenic capability of *E. coli*, *Salmonella enteritidis*, *Shigella dysenteriae*, and *Shigella flexneri* [227]. The anti-pathogenic potential of probiotics (*Lcb. rhamnosus*, *Lpb. plantarum*, *Lb. acidophilus*, and *Lcb. casei* strain Shirota) was strongly influenced by the matrix component [228].

### 10.1. Gut Microbiota and Gut–Brain Axis (GBA) Signaling

Furthermore, gut microbiota plays a key role in GBA signaling. Dysbiosis has been associated with alterations in GBA signaling and an increased risk of IBD [229]. Therefore, interventions that aim to restore a healthy gut microbiome, such as probiotics, prebiotics, and fecal microbiota transplantation, may also benefit the GBA and improve outcomes in patients with IBD [230]. Overall, research into the GBA in IBD is a rapidly evolving field with promising avenues for future treatment development. By better understanding the complex interactions between the gut and brain in IBD, one can identify novel therapeutic targets and improve patient outcomes [231].

### 10.2. Dietary Interventions Targeting the Gut Microbiome

Growing evidence suggests that the gut microbiome plays a key role in the pathogenesis of IBS and that diet and nutrition can modulate the composition and function of the microbiome [230]. The gut microbiome produces a variety of metabolites, including amino acids, biogenic amines and SCFAs, which can interact with host cells and influence gut physiology [35]. Recent studies have identified several biochemical pathways dysregulated in IBS, including those involved in immune function, intestinal permeability, and mucosal inflammation [230]. Diet and nutrition can modulate these pathways by altering the production of gut microbial metabolites, such as SCFAs, which have anti-inflammatory and immunomodulatory effects. For instance, a high-fiber diet has been shown to increase the production of SCFAs and improve symptoms in patients with IBS. These findings suggest that dietary interventions targeting the gut microbiome may be promising for managing IBS [230,231].

### 10.3. Probiotics as Medications

Probiotics and prebiotics can help alleviate symptoms associated with intestinal disorders such as IBD, IBS, and antibiotic-associated diarrhea [232]. They may reduce inflammation, promote regular bowel movements, and alleviate digestive discomfort [68]. The efficacy and specific benefits of probiotics, prebiotics, and symbiotics may vary depending on the individual, the specific strains or fibers used, and the dosage. When considering functional foods or supplements, it is recommended to consult with a physician or nutritionist who can provide personalized advice depending on health condition [233]. In terms of treatment, different medications are utilized in the management of IBD. 5-ASA (amino salicylates) accounts for 16% of treatment options. Thiopurines are utilized in a substantial percentage of cases (77.5%), followed by infliximab (25.4%) and adalimumab (15.3%), which are both anti-TNF biologic agents. Methotrexate, another medication option, is less frequently used (6.7%) [234].

### 10.4. Fecal Microbiota Transplantation (FMT)

There is an interplay between gut microbiota and inflammatory diseases. This insight may pave the way for the creation of probiotics and fecal microbiota transplantation; two targeted therapies improve the gut microbiota and the prognosis for people with inflammatory illnesses [235]. FMT, also known as stool transplantation or fecal bacteriotherapy, is a medical procedure in which the fecal matter from a healthy donor is transplanted into the gastrointestinal tract of a recipient. The purpose of FMT is to introduce a diverse and healthy microbial community into the recipient’s gut in order to restore or improve their gut microbiota [153,236]. The gut microbiota is a complex community of microorganisms that reside in the digestive tract, including bacteria, viruses, fungi, and other microbes. It plays a crucial role in maintaining gut health, digestion, immune function, and overall well-being. However, disruptions in the balance of the gut microbiota, such as due to antibiotic use, infections, or certain medical conditions, can lead to gastrointestinal disorders and other health problems. During an FMT procedure, the fecal material is carefully collected from a thoroughly screened and healthy donor. The fecal matter is processed, usually through dilution and filtration, to obtain a liquid suspension containing microbial components. This suspension is then administered to the recipient through various methods, such as colonoscopy, enema, nasogastric tube, or capsules. Once transplanted, the diverse microorganisms present in the donor feces can help restore the recipient’s gut microbiota by promoting a healthy microbial balance. The transplanted microbes can populate the recipient’s gut, establish themselves, and contribute to various beneficial functions, such as improving digestion, enhancing immune responses, and producing essential metabolites [237].

### 10.5. Microfluidic Technology

The novel microfluidic technology consists in microscale fluids using microchannels contained on a microfluidic chip [1,238]. It was first proposed in 1990 and has since undergone significant advances in complex manufacturing and interdisciplinary applications. Microfluidic technology mimics human intestinal health conditions and can assess associations with gut microbiota and for docking drug development and evaluation. Additionally, microfluidic drug delivery systems include drug pre-programming, delivery and applications with prebiotics, probiotics, and other active substances [239]. Microfluidic technology offers several advantages, such as simulating the gut normal functioning environment, co-cultivating multiple microorganisms with host cells, and delivering them for in-depth research [240]. Additionally, microfluidic drug delivery systems provide precise control over drug loading and production rates and can enhance the bioavailability and efficacy of bioactive contents. However, the technical threshold for using microfluidic drug delivery systems is high, requiring several components and affecting drug release efficiency [241]. Finally, we should emphasize that although there is great potential in the use of this technology, there are significant limitations and several challenges persisting in developing effective microfluidic-based methods, most of which focus on the complexities that arise from the nature (matrix) of samples and the need for seamless integration of different key steps on a single microfluidic chip.

## 11. Safety of Probiotics

Although probiotic strains are generally recognized as safe (GRAS status), this is not always the case due to adverse effects, poor quality of probiotic supplements and transmittable resistance to antibiotics [242].

Concerning the adverse effects of probiotics, one must stress the fact that there are numerous studies supporting the benefits of the usage of probiotics as health promoters while very few deal with the possible health hazards that they pose to healthy consumers and patients. As the absence of evidence is not equivalent to the evidence of absence, one must wonder about the validity of certain research protocols. Very few reports approach the modifications in structure and in function of the microbiomes before and after the administration of probiotics. Such approach reveals valuable information about the actual effect of probiotics and sheds light on their general mechanisms of action and on the idiosyncratic parameters of every individual patient [178,243].

Questions related to the safety of the supplements of probiotic products refer to purity, potency (as log CFU/g of final product), and ingredients of the final product. It is self-evident that probiotic products must undergo every necessary testing for potential contaminants [244,245,246]. Unwanted harmful microorganisms might contaminate the product.

The administration of probiotics is also another point of concern. Safe administration refers to the appropriate product and to the correct use and correct handling of the product, especially in hospital environments where circulating pathogens pose a serious risk for contamination [247].

Horizontal transfer of genes coding resistance to the antibiotic medicines from probiotic strains to pathogens in the intestines poses a serious risk [248]. This transfer via conjugation in vivo has been thoroughly documented and numerous reports verified the transferability of AR genes in the gut [249,250]. To assess the danger originating from the existence of AR genes within probiotic genomes, genotypic and phenotypic approaches are required. The most common method for the phenotypical approach is the minimum inhibitory concentration method (MIC), and in the interpretation of the results, the usual norm of the species should be taken into consideration [243,251]. Because normal AR ranges for many probiotic species have been established, any strain that exerts resistance beyond these limits must be further investigated [252]. For genotypic analysis, the complete genome sequence of the probiotic strain is necessary (the plasmids as well), and any genes coding AR should be identified. It follows that in the case that such genes are linked with mobile genetic elements, the strain should not be used in commercial supplements [253].

A very sporadic adverse effect of probiotic strains is associated with their translocation from the gastrointestinal tract causing invasive infection, particularly in children. This effect has been reviewed by D’agostin et al., where sepsis, bacteremia, and fungemia were associated with probiotic administration in children between 1995 and 2021, sepsis being the most common clinical condition. Most of the children with sepsis were beneath two years old with predisposing conditions such as prematurity or an intravenous catheter [254].

Intestinal microbiota affects, both directly and indirectly, the metabolism of drugs, intervening thus with their efficacy and toxicity [255,256]. This effect is possible through biochemical mechanisms such as decarboxylation (L-dopa), sulfation (acetaminophen), dehydroxylation (caffeic acid and L-dopa), demethylation (methamphetamine) and others [257,258]. Glucuronidation particularly might be important to the interaction between biodiversity of microbiomes, diet, and medicines [259].

## 12. Summarizing the Current and Future Perspectives

### 12.1. Clinical Research and Evidence

While there is growing evidence supporting the health benefits of certain probiotics, more robust clinical trials are needed. Large-scale, well-designed studies exploring the efficacy of probiotics for specific health conditions are necessary to provide clinicians and consumers with evidence-based recommendations. The quality, design, and methodology of studies investigating probiotics can vary significantly [260]. This heterogeneity makes it difficult to compare and draw definitive conclusions from the existing literature. More high-quality, well-designed studies with larger sample sizes are needed to establish robust evidence for specific probiotic strains and their effects. Addressing these challenges will contribute to advancing the field of probiotics, allowing for the development of targeted and effective interventions that can positively impact human health. Continued research, collaboration between academia and industry, and regulatory advancements are vital for unlocking the full potential of probiotics in the future.

### 12.2. Future Challenges and Limitations

While probiotics have gained significant popularity and recognition for their potential health benefits, there are several future challenges that researchers and the industry face in harnessing their full potential. Some of the key challenges and limitations are included below.

#### 12.2.1. Strain Specificity

Different strains of probiotic bacteria can have unique effects on the human body. Identifying the most effective strains for specific health conditions and understanding their mechanisms of action is crucial. Future research should focus on elucidating strain-specific effects and developing personalized probiotic interventions. Therefore, generalizing the effects of probiotics to all strains within a species may not be accurate. The efficacy of probiotics can also vary from person to person and depend on individual factors such as age, health status, diet, and gut microbiome composition [261]. What works for one individual may not work for another, and it can be challenging to predict the specific response to probiotic supplementation.

#### 12.2.2. Quality Control and Standardization

Ensuring the quality, viability, and stability of probiotic products is a challenge. Probiotics are live microorganisms, and maintaining their viability throughout production, storage, and consumption is essential. Establishing robust quality control measures and standardized guidelines for probiotic manufacturing is critical to ensure consistent and effective products.

#### 12.2.3. Survival in the Gastrointestinal Tract

Probiotics must survive the harsh conditions of the gastrointestinal tract to exert their beneficial effects. Improving the survival and colonization of probiotics in the gut is an ongoing challenge. Strategies such as microencapsulation, protective coatings, and genetic modification may enhance the survival rate of probiotic strains [262,263]. Probiotics are transient in the gut, and their effects may diminish once supplementation is stopped. They may not permanently colonize the gut microbiota in all individuals, and the benefits may cease once the probiotics are no longer consumed [263,264]. This highlights the need for continuous probiotic consumption to maintain their potential benefits. However, challenges such as exposure to stomach acid, bile salts, and competition from other gut microbes can impact their survival and viability. Ensuring that an adequate number of viable probiotic cells reach the intestines is crucial for their efficacy.

#### 12.2.4. Understanding Mechanisms of Action

Although probiotics have demonstrated various health benefits, more research is required to uncover the specific pathways and interactions between probiotics and the host. This knowledge will facilitate the development of targeted probiotic interventions.

#### 12.2.5. Personalized Approaches

Each individual has a unique gut microbiome composition and health status. Developing personalized, “tailor-made” probiotic interventions based on an individual’s microbiota profile, genetic makeup, and health conditions is crucial for optimizing outcomes [265]. Integrating metagenomic and meta/transcriptomic data, along with other personalized health information, can help tailor probiotic interventions to specific individuals.

To date, the knowledge of microbiome components is not being used in daily clinical practice, and validated tools for ecological descriptions are only available to researchers. Commercial offers of qualitative or quantitative measurements of the microbiota (“microbial profiles”) are currently not reliably interpretable and lack validated standards and references, ensuring their reproducibility and comparability.

Likewise, the results of fecal metabolite measurements from the microbiota (such as short-chain fatty acids, for example) are highly influenced by sampling and extraction conditions, resulting in heavily artifact-ridden interpretations of commercial kits in most cases [266].

#### 12.2.6. Regulatory Framework

The regulation of probiotics varies across different countries, and establishing clear guidelines and standards is necessary to secure common commercial and medical practices. Stricter regulations can ensure the safety, efficacy, and accurate labeling of probiotic products while still promoting innovation and development in the field.

## Figures and Tables

**Table 1:** Mixed approaches involved in reducing gut inflammation.

Term	Description
**Probiotics**	Microorganisms, when ingested in sufficient quantities, offer beneficial effects on host health.
**Pre-biotics**	Undegradable food ingredients that stimulate the growth of intestinal normal microbiota.
**Synbiotics**	A combination of probiotics and prebiotics that work synergistically for a healthy gut.

**Table 2:** Well-studied probiotic strains.

Genus	*Species*	Reference
***Lactobacillus***	*Lacticaseibacillus casei* (previously named *Lactobacillus casei*), *Lcb. rhamnosus*, *Lactobacillus acidophilus*, *Lactobacillus delbrueckii* subsp. *bulgaricus*, *Levilactobacillus brevis* (previously named *L. brevis*), *Lactobacillus delbrueckii* subsp. *lactis* (previously named *L. lactis*), *Lactiplantibacillus plantarum* subsp. *plantarum* (previously named *L. plantarum*), *Limosilactobacillus fermentum* (previously named *L. fermentum*)	[46,57,58]
***Bifidobacterium***	*B. bifidum*, *Bifidobacterium lactis*, *Bifidobacterium adolescentis*, *B. longum*, *B. breve*, *Bifidobacterium animalis*	[55,56,57,59]
***Saccharomyces***	*Saccharomyces boulardii*, *Saccharomyces cerevisiae M41*, *Saccharomyces cerevisiae B-18*	[48,60]
***Streptococcus***	*Streptococcus thermophilus*	[53,61,62]
***Escherichia***	*Escherichia coli Nissle 1917*	[63,64]
***Bacillus***	*Bacillus subtilis*	[65,66]
***Enterococcus***	*Enterococcus faecalis* *Enterococcus faecium*	[67,68]

**Table 3:** The beneficial and potential drawbacks of the normal enteric microbiota.

Positive Implications of Microbiota	Adverse Effects of Microbiota
**Bacterial competition**	Transformation of dietary procarcinogens into carcinogens
**Enhancement of mucosal immunity and preservation of mucosal integrity**	Intestinal dysbiosis disorders
**Sustaining peristalsis and metabolism of dietary carcinogens**	Opportunistic infection and gut-derived translocation
**Production of vitamin K and B complex**	
**Metabolism of prodrugs**	

**Table 4:** Metabolites that gut microbiota produces and their functions.

Metabolites	Functions	References
**Bile acid metabolites**	Glucose, lipid and energy metabolism, antimicrobial effects, signal transduction pathways.	[102,115]
**Phenolic derivatives**	Maintenance of intestinal health and protection against oxidative stress.	[83,116,117]
**Branched-chain fatty acids (BCFA)**	Increased histone acetylation.	[118,119]
**Indole derivatives**	Powerful antioxidant; regulation of intestinal barrier function.	[120,121]
**Ethanol**	Protein fermentation metabolite.	[122,123]
**Polyamines**	Intestinal barrier integrity and enhancement of specific immune system.	[14,124]
**Choline metabolites**	Regulation of lipid metabolism and glucose synthesis.	[125,126]
**Vitamin K and B complex**	Erythrocyte formation, DNA replication/repair, enzymatic co-factor.	[127,128]
**Hydrogen Sulfide (H_2_S)**	Neutralization of singlet reactive oxygen species.	[118,129,130]

**Table 5:** Desirable selection criteria of probiotics in food [215,216,217].

Parameters	Characteristics	Targeted Ways to Assess
**Safety**	Source of Virulence and Pathogenicity. Antibiotic resistance, toxicity, and metabolic activity are all variables in viral pathogenesis	Evaluation of the source or origin is important; for maximum effectiveness in the target species, it is preferable for the agent to have been isolated from within that species. For human consumption, probiotics derived from humans may be preferable. Constant monitoring both before and after release to the public
**Technological Acceptance**	Carrier foods have a high viability retention rate throughout the production and storage Organoleptic qualities that are of acceptable capacity for mass production Containing no phages	Research in vitro and the creation of new foods Model for sensory evaluation, finished goods, and consumer research on product development
**Functionality**	Ability to withstand acidic conditions and enzymes found in gastric juices Acceptance of bile Mucosal adherence and colonization consequences on health that have been shown and demonstrated	Effects on the stomach and bile have been studied using a variety of animal, in vitro, and human models Research on intestine segments, mucus, cell cultures, and animals/humans in vivo Clinical studies verify beneficial effects for health
**Desirable physiological criteria**	Immunomodulation Effects that are hostile to gastrointestinal pathogens Cancer-preventive and mutation-blocking qualities	Research on animals and people in labs and in the wild Pathogen adhesion and competitive exclusion in culture and animal models

## Data Availability

Not applicable.
